# *Sargassum* invasion in the Caribbean: the role of medical and scientific cooperation

**DOI:** 10.26633/RPSP.2019.52

**Published:** 2019-06-07

**Authors:** Dabor Resiere, Hossein Mehdaoui, Rémi Névière, Bruno Mégarbane

**Affiliations:** 1 University Hospital of Martinique University Hospital of Martinique Department of Critical Care Fort-de-FranceMartinique France Department of Critical Care, University Hospital of Martinique, Fort-de-France, Martinique, France; 2 Lariboisière Hospital, Paris-Diderot University Lariboisière Hospital, Paris-Diderot University Department of Medical and Toxicological Critical Care INSERM UMRS1144, Paris France Department of Medical and Toxicological Critical Care, Lariboisière Hospital, Paris-Diderot University, INSERM UMRS1144, Paris, France

Dear Editor:

Intensive oceanographic research to explain causes of the *Sargassum* seaweed invasion in the Caribbean should not overlook the potential risk of human airborne poisoning (1). Acute inhalation of hydrogen sulfide (H_2_S) produced by matter decomposition after 48 hours may result in potentially fatal hypoxic cardiac, respiratory and neurological failure. Chronic exposure to H2S may additionally produce neurological and cognitive impairments (2).

An alarming number of consultations and hospital admissions related to the effects of both acute and chronic exposures among the local population has been reported in the Caribbean since the beginning of the year 2018 (3). Along with environmental and economic preventive measures, the regional emergency plan addressing this enigmatic brown seaweed assault requires a public health perspective, including H_2_S level monitoring on affected beaches. This will require the training and deployment of doctors for monitoring, as well as experts in toxicology, in the involved areas (4).

There is a pressing requirement for the scientific community to understand the causes of these recent occurrences and to provide potential remedial solutions, as these invasions could have significant negative impacts on marine ecosystems, as well as economies including tourism, and healthcare programs for local Caribbean communities. In this context of climate change affecting the Caribbean islands, fight against *Sargassum* could become the catalyst for large- scale common ambitions. To answer the health and economic questions posed by *Sargassum* in a coherent way, implementing a common research-based long-term strategy appears to be an appealing idea

First, the effective protection of the populations against H_2_S emission from *Sargassum* decomposition requires the adequate knowledge of H_2_S and ammonia emissions, their degree of toxicities and effects on the environment (4). Secondly, regional medical cooperation is the only major asset to combat this scourge. Only by this way, we can bring tangible and lasting answers. Meetings between representatives of the Eastern Caribbean States (OECS), the Pan American

Health Organization (PAHO), the Caribbean Public Health Agency (CARPHA) and the Caribbean Community (CARICOM) are necessary to create common protocols to fight against *Sargassum*.

Health authorities need to work together to address this recurrent and extensive problem, which to date does not appear to have commenced. This problem affects many territories, and thus cannot be solved by individual country action. It is therefore imperative that the scientific community, both regionally and internationally, launch and maintain a joint cooperative approach to find solutions, as this phenomenon could travel well beyond our shores.

## Disclaimer.

The author holds sole responsibility for the views expressed in the manuscript, which may not necessarily reflect the opinion or policy of the *RPSP/PAJPH* and/or PAHO.

**Dabor Resiere** *   dabor.resiere@chu-martinique.fr

**Hossein Mehdaoui**

**Rémi Névière**

Department of Critical Care, University Hospital of Martinique, Fort-de-France, Martinique, France

**Bruno Mégarbane**

Department of Medical and Toxicological Critical Care, Lariboisière Hospital, Paris-Diderot University, INSERM UMRS1144, Paris, France
